# Prognostic value of plasma levels of HIF-1a and PGC-1a in breast cancer

**DOI:** 10.18632/oncotarget.12796

**Published:** 2016-10-21

**Authors:** Feng-Feng Cai, Cheng Xu, Xin Pan, Lu Cai, Xiao-Yan Lin, Su Chen, Ewelina Biskup

**Affiliations:** ^1^ Department of Breast Surgery, Yangpu Hospital, Tongji University School of Medicine, Shanghai, PR China; ^2^ Department of Central Laboratory, Yangpu Hospital, Tongji University School of Medicine, Shanghai, PR China; ^3^ Department of Molecular and Cellular Biology, School of Forensic Sciences, Xi'an Jiao Tong University Health Science Center, Xi'an, Shaanxi, PR China; ^4^ Department of Oncology, Department of Internal Medicine, University Hospital of Basel, Basel, Switzerland

**Keywords:** breast cancer, HIF-1α, PGC-1α, prognosis, ELISA

## Abstract

Cellular adaptive mechanisms are crucial for tumorigenesis and a common feature in solid tumor progression. Hypoxia-inducible factor-1α (HIF-1α) facilitates the biological response to hypoxia, advancing angiogenesis and metastatic potential of the tumor. The peroxisome proliferator–activated receptor γ coactivators 1α (PGC-1α) enhances mitochondrial biogenesis, favored by migratory/invasive cancer cells. We conducted a prospective, long-term follow up study to determine whether HIF-1α and PGC-1α can be implemented as predictive biomarker in breast cancer. HIF-1α and PGC-1α plasma concentrations were measured in patients and in healthy controls by enzyme linked immune sorbent assay. Breast cancer patients had significantly higher HIF-1α and PGC-1α levels, which correlated with clinicopathological features, overall with more aggressive cancer characteristics. Disease free and overall survival of breast cancer patients with high HIF-1α and PGC-1α were significantly poorer than in patients with low plasma levels. In multivariate analysis, high amount of PGC-1α showed independent prognostic value. Our data suggests that HIF-1α and PGC-1α may be promising, noninvasive, biomarkers with a high potential for future clinical implication to identify subgroups of patients with poorer prognosis and to indicate early, subclinical metastasis.

## INTRODUCTION

Scientific evidence confirms that tumor microenvironment influences tumorigenesis and tumor progression. Microenvironments of the vast majority of solid neoplasms, including breast cancer, are hypoxic [1, 2]. Hypoxia is a recognized event in cancer development, with a significant mutagenic potential [3]. Reciprocally, cancer itself induces hypoxia due to inflammatory processes, which activates a cascade of cytokines and chemokines [4–6]. Hypoxic cancer tissue microenvironment is thus closely related to tumor growth, tumor development, metastasis, therapy response and prognosis [7–10].

Cellular reaction to oxygen level is, in part, induced by hypoxia-inducible, oxygen-sensitive transcription factor HIF. This driving mediator consists of subunit proteins, which experience post-translational modifications in hypoxic conditions [11–13]. Hypoxia-inducible factor-1α (HIF-1α) is the functional subunit determining its activity [14, 15]. Under low concentrations of oxygen, HIF-1α is stabilized and its intracellular levels increase since ubiquitination and thus degradation is blocked [16].

Oncology research has been increasingly focusing on HIF-1α over the past decade. To date, it has been shown that HIF-1α activates more than one hundred target transcription genes, e.g. involved in glucose and high-energy phosphate transport and metabolism, erythropoiesis, etc. [17–19]. In that way, tumor cells maintain their metabolism and cellular energy level without an aerobic glycolysis (Warburg effect) [20–23]. This is different in acute hypoxia, where, glycolysis is initiated intracellularly at the substrate level (the Pasteur effect). HIF-1α also increases cytokines expression in chronic hypoxic microenvironment. Therefore, it is assumed that HIF-1α is facilitating carcinogenesis and tumor dissemination by promoting proliferation, angiogenesis, dedifferentiation and invasion. As hypoxia manifests itself, tumor cells express survival factors, resulting in resistance to radiation and chemotherapies [24]. HIF-1α is therefore the key player that allows cells to adapt to hypoxic conditions, and in case of tumor cells, to become less likely to respond to cytotoxic therapy. Last but not least, chronic hypoxia is associated with high microvessel density, causing vascular imbalance and poor perfusion and thus – together with tumor instability - metastasis and poor prognosis [14, 23, 25, 26]. HIF-1α may therefore present a novel diagnostic and therapeutic target. However, it has been insufficiently investigated in the most common noncutaneous cancer in females – the breast cancer.

Breast cancer is still causing most of cancer deaths in women, with a rising incidence along with demographical aging and manifestation of cancer risk factors in the modern lifestyle. Screening techniques remain a crucial part of prevention and reduction of cancer related deaths.

Data suggested that high HIF-1α-levels might be associated with more aggressive cancer characteristics. HIF-1α was even proposed as a prognostic marker, associated with a poor prognosis in selected patients' populations [27]. Despite controversial and partially divergent reports, HIF-1α seems to promote primary mammary tumor growth and metastasis [28, 29]. High HIF-1α levels are associated with proliferation and angiogenesis stimulated by VEGF [30, 31], and a shorter survival in lymph node negative breast cancer patients [32].

Similar hypotheses have been made for the peroxisome proliferator-activated receptor gamma coactivator 1-alph (PGC-1α) [33]. PGC-1α is a co-activator for steroid and nuclear receptors involved in energy metabolism, adaptive thermogenesis, fatty-acid oxidation, thyroid hormone receptors, cellular cholesterol homoeostasis and gluconeogenesis. Activation of these processes, along with an upregulated PGC-1α-expression, has been shown in brown adipose tissue under hypothermia [33–35]. Cancer cells prefer hypoxic conditions and adjust their metabolism to anabolic pathways. The mechanisms of tumor progression, proliferation, invasion and metastatic potential is still elusive, but recent data show that tumor cells in invasive tumors predominantly use mitochondrial respiration, where processes of oxidative phosphorylation are activated by PGC-1α [36]. Relation between PGC-1α and HIF-1α has been studied extensively and it seems that in angiogenesis, PGC-1α stimulates VEGF independently of HIF-1α. However, it might also be assumed that PGC-1α induced mitochondrial respiration lowers the oxygen level and increases ROS production [33]. Both conditions induce HIF-1α- activation. This has been shown in Birt-Hogg-Dubé (BHD) syndrome patients, who are at a higher risk to develop cancer. Molecularly, an increased mitochondrial metabolism is seen, due to AMPK activation at FLCN loss [37]. As a consequence, ROS number rises in the peri- and intracellular environment, which activates HIF [38, 39]. Also, new insights on the ERBB2 –gene revealed interesting pathway connections of PGC-1 and HIF-1: ERRα and PGC-1β have been suggested to stimulate the tumorigenesis of malignant breast cancer via regulation of ERBB2 expression [40]. Today we know that these processes influence glutamine enzyme expression in ERBB2+ breast cancer.

We addressed the critical question whether there is a reliability of HIF-1α and PGC-1α to be used as markers to predict prognosis in breast cancer patients. Most of the literature available on other cancer entities suggests that overexpression of HIF-1α is an indicator of poor prognosis, while some suggest contradictory results. PGC-1α has not been described in the context of predictive value for neoplasia. We aimed to study the protein PGC-1α as for several reasons: firstly, because there has been hypothesis made about PGC-1α expression being correlated to invasive nature of cancer cells, mammary gland tumorigenesis and formation of distant metastases [41]. However, PGC-1α role in a non-selected breast cancer population and its role as a biomarker have not been established yet. Secondly, based on the fact that HIF-1α and PGC-1α are sharing several cellular pathways, we investigated whether PGC-1α is also showing prognostic features in breast cancer.

Therefore, in our study, we aimed to investigate the role of HIF-1α, as well as PGC-1α in breast cancer tumorigenesis, growth and metastasis. We illustrated the correlation of both proteins' plasma levels in terms of the overall outcome and prognosis, based on a long term follow up of sampled breast cancer patients. Our data demonstrate that breast cancer patients have higher levels of both HIF-1α and PGC-1α. We studied the overexpression of both proteins separately and in combination as potential predictor of the therapy response and prognosis. We correlated the overexpression of HIF-1α and PGC-1α with patients' survival in an unselected population of breast cancer patients. We propose a simple and efficient measurement method of both proteins and discuss their predictive and therapeutic value. Our findings suggest a novel, improved clinical decision-making regarding adjuvant treatment of patients with breast carcinoma.

## RESULTS

### Patient characteristics

Altogether, we analyzed 297 female patients' plasma samples, including 267 patients with breast cancer and 30 patients with benign breast tumors. Median age was 56 years (range of 26 to 91 years old) in breast cancer and 39 years (range of 23 to 58 years old) in benign breast tumor group.

### Plasma levels of HIF-1α and PGC-1α in breast cancer and benign breast tumor patients

**Figure 1:**
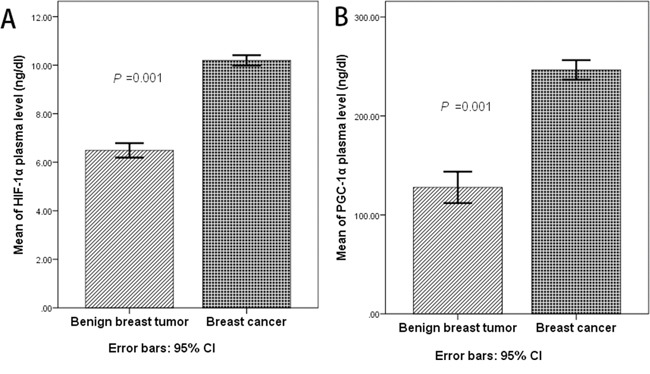
**A.** The mean of HIF-1α plasma level was 6.490ng/dl (95% CI 6.192-6.788) in benign breast tumor, while the mean of HIF-1α plasma level was 10.197ng/dl (95% CI 9.984-10.409) in breast cancer cohort (*P*=0.001, t' test); **B.** The mean of PGC-1α plasma level was 127.809ng/dl (95% CI 111.974-143. 644) in benign breast tumor, while the mean of PGC-1α plasma level was 246.502ng/dl (95% CI 236.606-256.400) in breast cancer cohort (*P*=0.001, t' test);

A total of 297 human plasma samples were assayed by ELISA to measure the plasma levels of HIF-1α and PGC-1α. In breast cancer cohort, mean HIF-1α plasma levels was 10.197ng/dl (95% CI 9.984-10.409), while the mean of PGC-1α plasma levels was 246.502ng/dl (95% CI 236.606-256.400). Both HIF-1α and PGC-1α plasma levels were significantly lower in benign breast tumor cohort, with a mean of HIF-1α plasma level 6.490ng/dl (95% CI 6.192-6.788), and 127.809ng/dl (95% CI 111.974-143. 644) for PGC-1α (Figure 1). In breast cancer cohort, both HIF-1α and PGC-1α plasma levels were closer to a normal distribution. The median of HIF-1α and PGC-1α plasma levels were used to conduct a dichotomy into patients with high HIF-1α and low HIF-1α plasma levels, as well as patients with high PGC-1α and low PGC-1α plasma levels. Following values were used: the median of HIF-1α plasma level 10.48 ng/dl, and the median of PGC-1α plasma level 244.24 ng/dl.

### Correlation of HIF-1α plasma level and clinicopathologic features

In the breast cancer cohort, we compared plasma level of HIF-1α with the clinicopathologic features, including the common survival predictors. There was a statistically significant correlation with multiple clinicopathologic characteristics: tumor size, skin involvement (edema, redness, nodularity or ulceration), lymph node metastasis and clinical stage (Table 1). The plasma level of HIF-1α in the invasive cancer group was higher than in ductal carcinoma in situ (DCIS) group. The data suggest that breast cancer patients with high HIF-1α plasma levels express more aggressive cancer characteristics and advanced stages. Nevertheless, patient's age, histologic grade, expression of hormone receptors and HER-2 have non-significant correlation with HIF-1α plasma levels.

**Table 1:** Clinicopathologic features and plasma level of HIF-1α

Category	No. of cases	plasma level of HIF-1α Mean± SD	Statistics value	*P* value
Age				
≤55	131	10.150±1.713	t =0.423	0.672
>55	136	10.242±1.817		
Tumor size				
≤2cm	164	9.698±1.795	t'= −6.599	0.001
>2cm	103	10.991±1.391		
Skin involvement^a^				
No	227	10.040±1.751	t = −3.532	0.001
Yes	40	11.086±1.585		
LN metastasis^b^				
No	169	9.728±1.675	F=14.853	0.001
1~3	49	11.079±1.387		
4~9	27	11.079±1.557		
10~	13	11.431±1.696		
Unknown	9			
Histologic grade				
≤II	206	10.167±1.812	t'= −1.895	0.061
>II	50	10.591±1.306		
Unknown	11			
Hormone receptor				
(−)	66	10.271±1.555	t = 0.274	0.785
(+)	188	10.201±1.859		
Unknown	13			
HER-2				
(−~+)	116	10.165±1.823	t= −0.758	0.450
(++~+++)	113	10.341±1.704		
Unknown	38			
Tumor type^c^				
DCIS^d^	26	8.646±2.101	Chi-Square =14.833	0.002
IDC^e^	224	10.380±1.636		
ILC^f^	5	10.258±2.879		
OTHER^g^	12	10.113±1.253		
Clinical stage^h^				
I	115	9.275±1.797	Chi-Square =43.153	0.001
II	83	10.729±1.221		
III	69	11.092±1.540		
PGC-1α plasma level^i^				
low	134	9.745±1.853	t'= −4.344	0.001
high	133	10.652±1.548		

a skin involvement includes: edema, redness, nodularity or ulceration of the skin

b Post Hoc Multiple Comparisons(LSD) shows difference of HIF-1αconcentration only presence in the group without LN metastasis and the other groups

c Post Hoc Multiple Comparisons(Games-Howell) shows difference of HIF-1αconcentration only presence in the DCIS group and the other groups

d DCIS: ductal carcinoma in situ

e IDC: invasive ductal carcinoma.

f ILC: invasive lobular carcinoma

g OTHER include: mucinous or colloid carcinoma, medullary carcinoma, metaplastic carcinoma.

h Post Hoc Multiple Comparisons(Games-Howell) shows difference of HIF-1αconcentration only presence in the stage I group and the other groups

i median of PGC-1α plasma level was 244.24 ng/dl

### Correlation of PGC-1α plasma level and clinicopathologic features

Further on, plasma level of PGC-1α was compared with different histopathological parameters. Analogously, the results showed that high plasma level of PGC-1α were closely related with larger tumor size, higher proportion of axillary lymph node metastasis, poorer histologic grade and advanced clinical stages, but not to patients' age, level of hormone receptor and HER-2 or skin involvement (Table 2). Similarly, invasive cancer group had higher plasma level of PGC-1α.

**Table 2:** Clinicopathologic features and plasma level of PGC-1α

Category	No. of cases	plasma level of PGC-1α Mean± SD	Statistics value	*P* value
Age				
≤55	131	248.882±79.446	t= −4.464	0.643
>55	136	244.211±84.863		
Tumor size				
≤2cm	164	227.298±84.600	t'= −5.293	0.001
>2cm	103	277.080±67.940		
Skin involvement^a^				
No	227	242.790±83.707	t = −1.767	0.078
Yes	40	267.572±69.757		
LN metastasis^b^				
No	169	235.066±85.258	F=4.231	0.006
1~3	49	261.348±73.066		
4~9	27	281.432±66.871		
10~	13	283.345±78.256		
Unknown	9			
Histologic grade				
≤II	206	242.499±80.471	t= −2.665	0.008
>II	50	276.416±81.712		
Unknown	11			
Hormone receptor				
(−)	66	248.723±93.207	t'=0.289	0.773
(+)	188	245.019±78.746		
Unknown	13			
HER-2				
(−~+)	116	237.449±72.534	t' = −0.956	0.340
(++~+++)	113	247.840±90.750		
Unknown	38			
Tumor type^c^				
DCIS^d^	26	202.973±81.314	F =3.191	0.024
IDC^e^	224	252.592±82.047		
ILC^f^	5	215.200±64.216		
OTHER^g^	12	240.260±66.221		
Clinical stage^h^				
I	115	216.291±83.529	F =15.275	0.001
II	83	266.301±75.351		
III	69	273.040±71.450		
HIF-1α plasma level^i^				
low	134	240.572±84.054	t = −1.185	0.237
high	133	252.477±80.014		

a skin involvement includes: edema, redness, nodularity or ulceration of the skin

b Post Hoc Multiple Comparisons(LSD) shows difference of PGC-1αconcentration only presence in the group without LN metastasis and the other groups

c Post Hoc Multiple Comparisons(LSD) shows difference of PGC-1αconcentration only presence in the DCIS group and the other groups

d DCIS: ductal carcinoma in situ

e IDC: invasive ductal carcinoma

f ILC: invasive lobular carcinoma

g OTHER include: mucinous or colloid carcinoma, medullary carcinoma, metaplastic carcinoma.

h Post Hoc Multiple Comparisons(LSD) shows difference of PGC-1αconcentration only presence in the stage I group and the other groups

i median of HIF-1α plasma level was 10.48 ng/dl

### Correlation between the plasma levels of HIF-1α and PGC-1α

Patients with a high PGC-1α plasma level also had a higher plasma level of HIF-1α. On the opposite, patients with a high HIF-1α plasma levels did not show higher plasma levels of PGC-1α (Table 1, 2).

### Plasma level of HIF-1α/ PGC-1α and survival

To evaluate HIF-1α- and PGC-1α-significance as clinical prognostic factors in breast cancer patients, we followed up all patients' groups and correlated their overall and disease free survival with HIF-1α/ PGC-1α levels. In a total, we followed-up 253 patients for a median of 42 months (range of 3 to 73 months). 79 patients suffered from tumor recurrence. By the end of follow-up period, 31 patients had died, 28 of breast cancer, and 174 patients had developed no recurrence.

**Figure 2:**
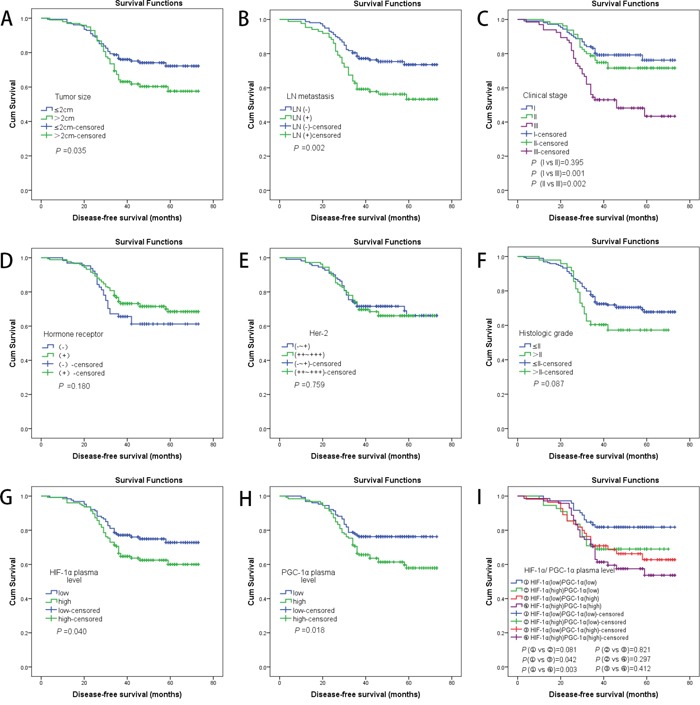
**A.** Kaplan–Meier analyses of the effect tumor size on DFS (*P* =0.035, log-rank test); **B.** Kaplan-Meier analyses of the effect LN metastasis on DFS (*P* =0.002, log-rank test); **C.** Kaplan-Meier analyses of the effect clinical stage on DFS (log-rank test shows difference of DFS only presence in the stage III group and the other groups); **D.** Kaplan-Meier analyses of the effect hormone receptor on DFS (*P* =0.180, log-rank test); **E.** Kaplan-Meier analyses of the effect HER-2 on DFS (*P* =0.759, log-rank test); **F.** Kaplan-Meier analyses of the effect histologic grade on DFS (*P* =0.087, log-rank test); **G.** Kaplan-Meier analyses of the effect HIF-1α plasma level on DFS (*P* =0.040, log-rank test); **H.** Kaplan-Meier analyses of the effect PGC-1α plasma level on DFS (*P* =0.018, log-rank test); **I.** Kaplan-Meier analyses of the effect HIF-1α and PGC-1α plasma level on DFS (log-rank test shows difference of DFS only presence in the group ① and the groups ③/④);

We arbitrarily divided all patients into two groups by median of HIF-1α/ PGC-1α plasma levels. The median of HIF-1α plasma level was 10.48 ng/dl, and the median of PGC-1α plasma level was 244.24 ng/dl. As shown in Figure 2G and 2H, patients with lower HIF-1α/ PGC-1α plasma levels (under the median value) had a better DFS compared to those with high HIF-1α/ PGC-1α plasma levels. The difference between the two groups was statistical significantly (*P* < 0.05, log-rank test). We conducted an analogous control, as shown in Figures 2A, 2B and 2C. Cancer patients' group with adverse prognostic factors (such as larger tumor size, axillary lymph node metastasis) had a significantly shorter DFS than patients without these factors among 253 patients (*P* < 0.05, log-rank test). As indicated by DFS curves constructed for the comparison of four different groups based on survival results in correlation to HIF-1α and PGC-1α plasma levels, the prognosis in patients with both lower plasma level of HIF-1α and PGC-1α was better than that of those with high PGC-1α, independently of HIF-1α changes (Figure 2I).

**Figure 3:**
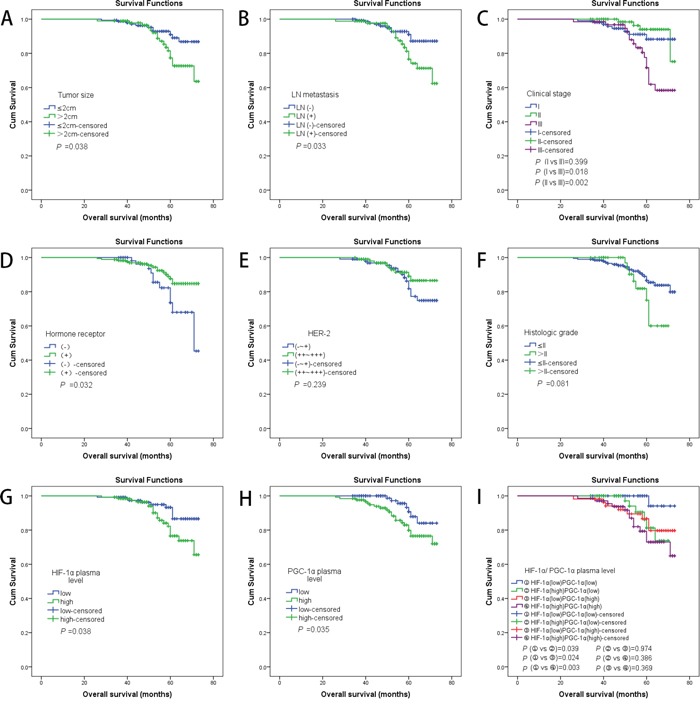
**A.** Kaplan–Meier analyses of the effect tumor size on OS (*P* =0.038, log-rank test); **B.** Kaplan-Meier analyses of the effect LN metastasis on OS (*P* =0.033, log-rank test); **C.** Kaplan-Meier analyses of the effect clinical stage on OS (log-rank test shows difference of OS only presence in the stage III group and the other groups); **D.** Kaplan-Meier analyses of the effect hormone receptor on OS (*P* =0.032, log-rank test); **E.** Kaplan-Meier analyses of the effect HER-2 on OS (*P* =0.239, log-rank test); **F.** Kaplan-Meier analyses of the effect histologic grade on OS (*P* =0.081, log-rank test); **G.** Kaplan-Meier analyses of the effect HIF-1α plasma level on OS (*P* =0.038, log-rank test); **H.** Kaplan-Meier analyses of the effect PGC-1α plasma level on OS (*P* =0.035, log-rank test); **I.** Kaplan-Meier analyses of the effect HIF-1α and PGC-1α plasma level on OS (log-rank test shows difference of OS only presence in the group ① and the other groups);

The results of overall survival analysis are corresponding to the results of DFS (Figure 3). These results clearly indicated a statistically significant correlation between high HIF-1α/ PGC-1α plasma levels and poorer survival outcomes.

### PGC-1α plasma level is an independent prognostic factor for disease free survival in breast cancer patients

In a multivariate COX 's Proportional Hazard Model, axillary lymph node metastasis and hormone receptor negativity were independent factors for a poorer disease free survival. Presence of axillary lymph node metastasis seemed to influence the disease free survival more significantly (HR=1.990, 95.0% CI 1.204~3.289, *P* < 0.01, Table 3).

**Table 3:** Multivariate Analyses of DFS (Backward Stepwise, Likelihood Ratio)

Variable	HR	95%CI	*P* value^a^
Tumor size			
(≤2cm vs >2cm)	1.089	0.645 to 1.840	0.749
LN metastasis			
(No vs Yes)	1.990	1.204 to 3.289	0.007
Histologic grade			
(≤II vs >II)	1.470	0.797 to 2.711	0.217
Hormone receptor			
(negative vs positive)	0.594	0.349 to 1.011	0.055
HER-2			
(−~+ vs ++~+++)	1.015	0.610 to 1.687	0.955
HIF-1α plasma level			
(low vs high)	1.429	0.861 to 2.373	0.167
PGC-1α plasma level			
(low vs high)	1.582	0.951 to 2.633	0.077

a *P* values less than 0.1 were considered as statistically significant in multivariate analyses

Most strikingly, in our multivariate analysis, which was also employed to evaluate the clinical impact of HIF-1α/ PGC-1α plasma level on prognosis, PGC-1α plasma level was an independent prognostic factor for disease free survival in breast cancer patients (HR=1.582, 95.0% CI 0.951~2.633, *P* < 0.1).

## DISCUSSION

Current markers to predict prognosis of breast cancer patients are insufficient and still based on quite unspecific clinicopathological features such as HR status, TNM classification and tumor grading [42, 43]. However, breast cancer is an extremely complex, heterogenic and alterable disease. Patients in the same stage still show great differences in their therapy response and survival. Individual and personalized approaches for diagnosis, therapy and follow up, as well as molecular parameters that can assist selecting high risk patients, monitor them along the therapy course and detect recurrences at a subcellular level, are emergently needed.

Hypoxia is well known to be associated with tumor environment and is a vague prognostic marker [21]. Under hypoxic conditions, the cell adapts to stress by stabilizing and upregulating HIF-1α is, a crucial transcriptive factor regulating diverse biological functions and activating critical genes involved in angiogenesis, migration, invasion, and metastasis [44–46]. Also in breast cancer, hypoxia is omnipresent and has been described in large cohort studies as associated to increased risk of metastasis, relapse, and mortality. The median *P*O2 in normal breast tissue is 65 mmHg. In breast cancer patients it is estimated at 10 mmHg (~1.5% O2).

Besides of the mechanisms mentioned above, HIF-1α stimulates the breast cancer stem cell phenotype (BCSC phenotype), which possesses the ability to form metastatic and recurrent tumors by stimulating hypoxic breast cancer cell motility. In various cancer entities, hypoxia has been reported related to insensitivity to chemotherapy and radiotherapy, higher tumor aggressivity, invasiveness and a poor prognosis.

The clinical implication of HIF-1α levels in tumors is controversial. Data showed that HIF-1α overexpression is correlated with poorer survival in oligodendroglioma, endometrial, uterine cervical, ovarian, esophageal, lung, head and neck cancer. So far, only several reports looked at HIF-1α plasma level in breast cancer as a prognostic factor. Bos and colleagues investigated a selected population, where locally advanced tumors have been excluded. Increased HIF-1α levels were associated with a poor OS and DFS in node-negative, but not in node-positive patients [32]. In Schindl's and Gruber's studies, survival correlations were confirmed for node-positive patients [27, 47]. The latter findings are in agreement with our results, which have a stronger power since we included an unselected patients' cohort and we had a significantly higher n-number.

Some researches demonstrated that the plasma level of HIF-1α is high in breast cancer patients. The technology used to draw these conclusions should be seen from a scientific perspective: most of the available studies are based on immunohistochemistry. More accurate methods, such as ELISA, provide more reliable results.

PGC-1α has gained an illicit interest nowadays, since it is potentially associated with cancer [48]. It is involved in cells diverting their metabolism into anabolic pathways, which is indirectly linked to tumorigenesis and tumor growth. Increased levels of PGC-1 family coactivators have been observed in cancer cells. Recently, it has been shown that specifically invasive cancer cells use mitochondrial respiration, in which PGC-1α is a key upregulator of oxidative phosphorylation [49–51]. These processes are of a great importance for a proper mobility of tumor cells, and thus their ability to migrate [52, 53]. It was therefore assumable that there is a relation between PGC-1α level and metastases. Altogether, PGC-1α is associated with suppression of apoptosis and metabolic reprogramming [54, 55]. However, the role of the PGC-1α appears to be complex, several studies revealed a significantly decreased PGC-1α coactivator expression in tumor samples, while a forced expression of PGC-1α even resulted in apoptosis of human epithelial ovarian cancer cells [56, 57].

According to our results, plasma levels of both proteins in patients with breast cancer were significantly higher than benign breast tumor patients (*P* = 0.001). We found a significant correlation between plasma levels of both proteins and patients' clinicopathological features. High levels of HIF-1α and PGC-1α were both related to more aggressive types of breast cancer. This suggests that HIF-1α and PGC-1α may stimulate tumor development and metastasis, and to have power to predict the prognosis in breast cancer patients.

Survival data of patients were collected and patients were followed-up. Correlation with the plasma levels of HIF-1α and PGC-1α were analyzed. We established a cut off and divided patients into HIF-1α/PGC-1α high and HIF-1α/PGC-1α low groups. We found that the subgroup with HIF-1α and PGC-1α high plasma level was significantly associated with a poorer survival. The survival of patients with breast cancer whose plasma level of HIF-1α was lower than median was much greater than the survival of patients whose plasma level of HIF-1α was higher than the median (*P*=0.001). In multivariate analysis, PGC-1α has been statistically proven to be an independent prognostic factor for the outcome in breast cancer patients.

These findings correlate with the hypothesis that hypoxia induces modifications of cells, which are then equipped for survival under adverse microenvironmental conditions. Most of these changes are driven by HIF-1α and PGC-1α. As a result, cells achieve an enhanced metastatic and a reduced apoptotic potential. This in return leads to resistance to cytotoxic therapies, especially radiotherapy. In our study, HIF-1α and PGC-1α were measured in all breast cancer patients. In our cohort, patients with a high PGC-1α plasma level also had a higher plasma level of HIF-1α, but not vice versa. These results, along with PGC-1α plasma level being an independent prognostic factor for DFS in an unselected breast cancer patients' population, suggest that PGC-1α is the key factor in hypoxia induced modifications of breast cancer cells. HIF-1α high patients who were PGC-1α positive did not differ from PGC-1α negative patients with respect to clinico-pathological characteristics. This said, PGC-1α is potentially a much more solid and stronger marker with a more reliable prognostic value than HIF-1α.

Therapeutic approaches that target HIF-1α and PGC-1α pathways are to be elaborated, as they seem promising. Preeliminary *in vitro* data showed synergistic effects of HIF-1α antisense agents and cytotoxic drugs.

Along with the tumor progression, metastasis and during anticancer therapy, molecular changes result in various constellation of potential marker proteins. The strength of our study is a comparatively large study population of not pretreated patients and a long term follow up. Moreover, we used a simple and exact quantification method of ELISA. Using this method allowed us to rely on objective numeric values and not on biased immunohistochemistry-based observations on HIF-1α/PGC-1α amounts. Additionally, we have used patients' plasma as detection material. Obviously, this sampling is easier and more feasible than tumor tissues. Altogether, we suggest a novel, easy and effective way to assess HIF-1α and PGC-1α-levels in the plasma.

There were some limitations of our study. The investigated patient cohort is not that large and the observed survival period not that long; further studies are needed to validate these findings in order to enable a translation of our results into clinical decision-making. Since the adjuvant therapy has a significant impact on the outcome, it would be ideal to have all patients treated with exact same protocol. However, since current standard adjuvant therapies are similar in their efficiency, and since all our patients were treated according to the IA recommended guidelines, it can be assumed that the influence of the chosen adjuvant treatment was comparable and statistically indifferent for the entire study group.

There are still remaining open questions, which we will approach shortly. Firstly, we would like to determine whether both proteins could become diagnostic markers in breast cancer. While HIF-1α has not reached an independent prognostic marker status in our study and in most of previous trials, it is doubtable that it will be of an imminent value for diagnostics in breast cancer. Potentially, HIF-1α plasma level can fluctuate, especially in patients with chronic inflammatory diseases, so that even a very large control group will hardly neutralize the biological variation bias in trials.

PGC-1α however seems to be more sensitive and shows promise in having a diagnostic value, with an easy and efficient methodology, using routine blood test. It is conceivable that pattern of PGC-1α expression is sufficiently stable and invariable at a very early, preclinical stage of breast cancer, and thus serve as a reliable diagnostic signature. At this moment, randomized, prospective trials are missing to make representative conclusions.

Investigations of whether a dynamic monitoring of HIF-1α and PGC-1α plasma levels could reflect the therapy response along a related treatment are another open aspect that deserves scientific attention. Reciprocally, it would be interesting to determine whether concentrations of HIF-1α and PGC-1α increase during disease progression, since it would allow a simple serological test to identify therapy resistant or relapsed cases.

Furthermore, it would be important to establish whether the use of specific HIF-1α and PGC-1α inhibitors could achieve a curative effect when used in patients with high HIF-1α and PGC-1α levels. Also, since both proteins are associated with metastatic potential of cancer cells, it would be crucial to elaborate whether plasma levels monitoring would allow for indications of early, subclinical metastasis processes.

Taken together, we found that HIF-1α and PGC-1α overexpression has prognostic significance in breast cancer patients and may present potential opportunities in breast cancer therapy. We identified easily detectable, non-invasive new biological markers with predictive power. Our results suggest the manner of how we should choose suitable patient groups to apply HIF-1α and PGC-1α inhibitors. Testing of HIF-1α and PGC-1α plasma level scan assist the clinical decision-making and evaluate the treatment efficacy on an individual, personalized basis.

## MATERIALS AND METHODS

### Study population

Blood samples used in this study were collected in a time period from June 2009 to June 2014 in the Department of Breast Surgery, Yangpu Hospital, Tongji University School of Medicine.

We prospectively and randomly recruited 297 patients (267 breast cancer patients and 30 benign breast tumor patients), establishing associated clinicopathologic database. Long-term clinical follow up of 42 months (range of 3 to 73 months) was available for 253 patients. 79 patients suffered from tumor recurrence. By the end of follow-up period, 31 patients had died. In 28 patients the cause of death was breast cancer. 174 patients developed no recurrence.

Prior to collecting plasma samples, no neoadjuvant treatments of any type were permitted. All patients were Chinese females and were followed until death or the end of the follow-up period.

### Collecting and dealing with the samples

5 ml of fasting blood sample were drawn from antecubital vein of every breast cancer and benign tumor patients. The blood samples were put into the EDTA anticoagulative tube, centrifuged for ten minutes (1000 r/min) to separate plasma and stored in −80°C for further analysis.

### Ethics statement

The ethics review board approved the study design a priori. The protocol was approved by the Ethics Committee of Yangpu Hospital, Tongji University School of Medicine. Written informed consent was obtained from each patient. The method described in this study, including acquisition of blood samples, was carried out in accordance with the approved guidelines and regulations.

### Detection of HIF-1α and PGC-1α by ELISA

ELISA is an efficient and effective method in order to assess the expression level of HIF-1α and PGC-1α in plasma samples. A total of 297 human plasma samples were assayed by ELISA The concentration of HIF-1α and PGC-1α in breast cancer and benign tumor patients were determined using ELISA kits according to the manufacturer's instructions (Human hypoxia-inducible factor 1α ELISA kit, DRE10248, Shanghai bioleaf biotech Co., Ltd. Shanghai, China; Human peroxisome proliferator activated receptor gamma coactivator 1α ELISA kit, DRE11477, Shanghai bioleaf biotech Co., Ltd. Shanghai, China). The sample dilution used for diluting plasma was analyzed as a blank control. The optical density (OD) of each well was measured at a wavelength of 450 nm in Varioskan Flash Multimode reader (Thermo Scientific, Waltham, Massachusetts, US). The concentration of HIF-1α and PGC-1α were calibrated with the HIF-1α and PGC-1α standard curve. Assays were repeated in duplicate.

### Statistical analysis

The correlation between plasma levels of HIF-1α or PGC-1α and clinicopathologic features were analyzed using T test or ANOVA. Disease-free survival (DFS) was defined as the period from the operative date to the first recurrence (local or distant) or death of breast cancer without a recorded relapse. Overall survival (OS) was defined as the period from the operative date to death of breast cancer. The survival curves of each group were estimated by Kaplan-Meier survival analyses, and the curves were analyzed by the log-rank test. In the multivariate analysis, a COX's Proportional Hazard Model was employed to estimate whether a factor was a significant independent prognostic factor of survival. All statistical tests were two-sided, *P* values less than 0.05 were considered as statistically significant in univariate analysis, and *P* values less than 0.1 were considered as statistically significant in multivariate analysis. The statistical analyses were performed using SPSS 22.0 software (SPSS Inc.).

## References

[1] Lu H, Ouyang W, Huang C (2006). Inflammation, a key event in cancer development. Mol Cancer Res.

[2] Rakoff-Nahoum S (2006). Why cancer and inflammation?. Yale J Biol Med.

[3] Greijer AE, van der Wall E (2004). The role of hypoxia inducible factor 1 (HIF-1) in hypoxia induced apoptosis. J Clin Pathol.

[4] Balkwill F, Mantovani A (2001). Inflammation and cancer: back to Virchow?. Lancet.

[5] Coussens LM, Werb Z (2002). Inflammation and cancer. Nature.

[6] Aggarwal BB, Shishodia S, Sandur SK, Pandey MK, Sethi G (2006). Inflammation and cancer: how hot is the link?. Biochem Pharmacol.

[7] Grivennikov SI, Greten FR, Karin M (2010). Immunity, inflammation, and cancer. Cell.

[8] Candido J, Hagemann T (2013). Cancer-related inflammation. J Clin Immunol.

[9] Mantovani A, Allavena P, Sica A, Balkwill F (2008). Cancer-related inflammation. Nature.

[10] Allavena P, Germano G, Marchesi F, Mantovani A (2011). Chemokines in cancer related inflammation. Exp Cell Res.

[11] Bilton RL, Booker GW (2003). The subtle side to hypoxia inducible factor (HIFalpha) regulation. Eur J Biochem.

[12] Ivan M, Kondo K, Yang H, Kim W, Valiando J, Ohh M, Salic A, Asara JM, Lane WS, Kaelin WG (2001). HIFalpha targeted for VHL-mediated destruction by proline hydroxylation: implications for O2 sensing. Science.

[13] Kaelin WG (2005). Proline hydroxylation and gene expression. Annu Rev Biochem.

[14] Zhang W, Shi X, Peng Y, Wu M, Zhang P, Xie R, Wu Y, Yan Q, Liu S, Wang J (2015). HIF-1alpha Promotes Epithelial-Mesenchymal Transition and Metastasis through Direct Regulation of ZEB1 in Colorectal Cancer. PLoS One.

[15] Kimbro KS, Simons JW (2006). Hypoxia-inducible factor-1 in human breast and prostate cancer. Endocr Relat Cancer.

[16] Hackenbeck T, Knaup KX, Schietke R, Schodel J, Willam C, Wu X, Warnecke C, Eckardt KU, Wiesener MS (2009). HIF-1 or HIF-2 induction is sufficient to achieve cell cycle arrest in NIH3T3 mouse fibroblasts independent from hypoxia. Cell Cycle.

[17] Shaw RJ (2006). Glucose metabolism and cancer. Curr Opin Cell Biol.

[18] Tomlinson DR, Gardiner NJ (2008). Glucose neurotoxicity. Nat Rev Neurosci.

[19] Ginouves A, Ilc K, Macias N, Pouyssegur J, Berra E (2008). PHDs overactivation during chronic hypoxia “desensitizes” HIFalpha and protects cells from necrosis. Proc Natl Acad Sci U S A.

[20] Ke Q, Costa M (2006). Hypoxia-inducible factor-1 (HIF-1). Mol Pharmacol.

[21] Brocato J, Chervona Y, Costa M (2014). Molecular responses to hypoxia-inducible factor 1alpha and beyond. Mol Pharmacol.

[22] Elson DA, Ryan HE, Snow JW, Johnson R, Arbeit JM (2000). Coordinate up-regulation of hypoxia inducible factor (HIF)-1alpha and HIF-1 target genes during multi-stage epidermal carcinogenesis and wound healing. Cancer Res.

[23] Cui H, Grosso S, Schelter F, Mari B, Kruger A (2012). On the Pro-Metastatic Stress Response to Cancer Therapies: Evidence for a Positive Co-Operation between TIMP-1, HIF-1alpha, and miR-210. Front Pharmacol.

[24] Mathieu J, Zhang Z, Zhou W, Wang AJ, Heddleston JM, Pinna CM, Hubaud A, Stadler B, Choi M, Bar M, Tewari M, Liu A, Vessella R (2011). HIF induces human embryonic stem cell markers in cancer cells. Cancer Res.

[25] Calzada MJ, del Peso L (2007). Hypoxia-inducible factors and cancer. Clin Transl Oncol.

[26] Chung J, Roberts AM, Chow J, Coady-Osberg N, Ohh M (2006). Homotypic association between tumour-associated VHL proteins leads to the restoration of HIF pathway. Oncogene.

[27] Gruber G, Greiner RH, Hlushchuk R, Aebersold DM, Altermatt HJ, Berclaz G, Djonov V (2004). Hypoxia-inducible factor 1 alpha in high-risk breast cancer: an independent prognostic parameter?. Breast Cancer Res.

[28] Schwab LP, Peacock DL, Majumdar D, Ingels JF, Jensen LC, Smith KD, Cushing RC, Seagroves TN (2012). Hypoxia-inducible factor 1alpha promotes primary tumor growth and tumor-initiating cell activity in breast cancer. Breast Cancer Res.

[29] Liu ZJ, Semenza GL, Zhang HF (2015). Hypoxia-inducible factor 1 and breast cancer metastasis. J Zhejiang Univ Sci B.

[30] Bos R, Zhong H, Hanrahan CF, Mommers EC, Semenza GL, Pinedo HM, Abeloff MD, Simons JW, van Diest PJ, van der Wall E (2001). Levels of hypoxia-inducible factor-1 alpha during breast carcinogenesis. J Natl Cancer Inst.

[31] Isobe T, Aoyagi K, Koufuji K, Shirouzu K, Kawahara A, Taira T, Kage M (2013). Clinicopathological significance of hypoxia-inducible factor-1 alpha (HIF-1alpha) expression in gastric cancer. Int J Clin Oncol.

[32] Bos R, van der Groep P, Greijer AE, Shvarts A, Meijer S, Pinedo HM, Semenza GL, van Diest PJ, van der Wall E (2003). Levels of hypoxia-inducible factor-1alpha independently predict prognosis in patients with lymph node negative breast carcinoma. Cancer.

[33] Puigserver P (2005). Tissue-specific regulation of metabolic pathways through the transcriptional coactivator PGC1-alpha. Int J Obes (Lond).

[34] Meirhaeghe A, Crowley V, Lenaghan C, Lelliott C, Green K, Stewart A, Hart K, Schinner S, Sethi JK, Yeo G, Brand MD, Cortright RN, O'Rahilly S, Montague C, Vidal-Puig AJ (2003). Characterization of the human, mouse and rat PGC1 beta (peroxisome-proliferator-activated receptor-gamma co-activator 1 beta) gene in vitro and in vivo. Biochem J.

[35] Patti ME, Butte AJ, Crunkhorn S, Cusi K, Berria R, Kashyap S, Miyazaki Y, Kohane I, Costello M, Saccone R, Landaker EJ, Goldfine AB, Mun E (2003). Coordinated reduction of genes of oxidative metabolism in humans with insulin resistance and diabetes: Potential role of PGC1 and NRF1. Proc Natl Acad Sci U S A.

[36] LeBleu VS, O'Connell JT, Gonzalez Herrera KN, Wikman H, Pantel K, Haigis MC, de Carvalho FM, Damascena A, Domingos Chinen LT, Rocha RM, Asara JM, Kalluri R (2014). PGC-1alpha mediates mitochondrial biogenesis and oxidative phosphorylation in cancer cells to promote metastasis. Nat Cell Biol.

[37] Welsch MJ, Krunic A, Medenica MM (2005). Birt-Hogg-Dube Syndrome. Int J Dermatol.

[38] Yan M, Gingras MC, Dunlop EA, Nouet Y, Dupuy F, Jalali Z, Possik E, Coull BJ, Kharitidi D, Dydensborg AB, Faubert B, Kamps M, Sabourin S (2014). The tumor suppressor folliculin regulates AMPK-dependent metabolic transformation. J Clin Invest.

[39] Bhalla K, Hwang BJ, Dewi RE, Ou L, Twaddel W, Fang HB, Vafai SB, Vazquez F, Puigserver P, Boros L, Girnun GD (2011). PGC1alpha promotes tumor growth by inducing gene expression programs supporting lipogenesis. Cancer Res.

[40] Deblois G, Chahrour G, Perry MC, Sylvain-Drolet G, Muller WJ, Giguere V (2010). Transcriptional control of the ERBB2 amplicon by ERRalpha and PGC-1beta promotes mammary gland tumorigenesis. Cancer Res.

[41] O'Hagan KA, Cocchiglia S, Zhdanov AV, Tambuwala MM, Cummins EP, Monfared M, Agbor TA, Garvey JF, Papkovsky DB, Taylor CT, Allan BB (2009). PGC-1alpha is coupled to HIF-1alpha-dependent gene expression by increasing mitochondrial oxygen consumption in skeletal muscle cells. Proc Natl Acad Sci U S A.

[42] Pace LE, Keating NL (2014). A systematic assessment of benefits and risks to guide breast cancer screening decisions. JAMA.

[43] Payne SJ, Bowen RL, Jones JL, Wells CA (2008). Predictive markers in breast cancer--the present. Histopathology.

[44] Hanahan D, Weinberg RA (2011). Hallmarks of cancer: the next generation. Cell.

[45] Cavallo F, De Giovanni C, Nanni P, Forni G, Lollini PL (2011). 2011: the immune hallmarks of cancer. Cancer Immunol Immunother.

[46] Lazebnik Y (2010). What are the hallmarks of cancer?. Nat Rev Cancer.

[47] Schindl M, Schoppmann SF, Samonigg H, Hausmaninger H, Kwasny W, Gnant M, Jakesz R, Kubista E, Birner P, Oberhuber G, Austrian B, Colorectal Cancer Study G (2002). Overexpression of hypoxia-inducible factor 1alpha is associated with an unfavorable prognosis in lymph node-positive breast cancer. Clin Cancer Res.

[48] Jones AW, Yao Z, Vicencio JM, Karkucinska-Wieckowska A, Szabadkai G (2012). PGC-1 family coactivators and cell fate: roles in cancer, neurodegeneration, cardiovascular disease and retrograde mitochondria-nucleus signalling. Mitochondrion.

[49] Savagner F, Mirebeau D, Jacques C, Guyetant S, Morgan C, Franc B, Reynier P, Malthiery Y (2003). PGC-1-related coactivator and targets are upregulated in thyroid oncocytoma. Biochem Biophys Res Commun.

[50] Soyal S, Krempler F, Oberkofler H, Patsch W (2006). PGC-1alpha: a potent transcriptional cofactor involved in the pathogenesis of type 2 diabetes. Diabetologia.

[51] Shiota M, Yokomizo A, Tada Y, Inokuchi J, Tatsugami K, Kuroiwa K, Uchiumi T, Fujimoto N, Seki N, Naito S (2010). Peroxisome proliferator-activated receptor gamma coactivator-1alpha interacts with the androgen receptor (AR) and promotes prostate cancer cell growth by activating the AR. Mol Endocrinol.

[52] Orrenius S, Zhivotovsky B, Nicotera P (2003). Regulation of cell death: the calcium-apoptosis link. Nat Rev Mol Cell Biol.

[53] Duchen MR, Verkhratsky A, Muallem S (2008). Mitochondria and calcium in health and disease. Cell Calcium.

[54] DeBerardinis RJ, Lum JJ, Hatzivassiliou G, Thompson CB (2008). The biology of cancer: metabolic reprogramming fuels cell growth and proliferation. Cell Metab.

[55] Boroughs LK, DeBerardinis RJ (2015). Metabolic pathways promoting cancer cell survival and growth. Nat Cell Biol.

[56] Jiang WG, Douglas-Jones A, Mansel RE (2003). Expression of peroxisome-proliferator activated receptor-gamma (PPARgamma) and the PPARgamma co-activator, PGC-1, in human breast cancer correlates with clinical outcomes. Int J Cancer.

[57] Zhang Y, Ba Y, Liu C, Sun G, Ding L, Gao S, Hao J, Yu Z, Zhang J, Zen K, Tong Z, Xiang Y, Zhang CY (2007). PGC-1alpha induces apoptosis in human epithelial ovarian cancer cells through a PPARgamma-dependent pathway. Cell Res.

